# Women and the Baltimore Fire

**Published:** 1904-06

**Authors:** 


### WOMEN AND THE BALTIMORE FIRE.

A generous and graceful tribute to the calmness of women in danger is that paid by Mr. Lynn Roby Meekins, editor of the Baltimore Herald, in his article in the June *Delineator.* He tells not only of women who worked in the thick of the destruction with and for their employers' husbands and fathers, and of the brave and cheerful way in which, when all was lost, they set to work at humble occupations to support themselves, but of their ability to rise quite above the consideration of material things in the recognition of essentials. "One young husband," he says, "made his way home and, pale and trembling, approached his wife. 'I am ruined,' he exclaimed. 'My dear,' she replied, smiling through her anxiety, 'you are not hurt a bit.' That was the woman's view of the situation. It was mainly personal."

